# Co‐MnO_2_ Nanorods for High‐Performance Sodium/Potassium‐Ion Batteries and Highly Conductive Gel‐Type Supercapacitors

**DOI:** 10.1002/advs.202105510

**Published:** 2022-01-27

**Authors:** Jun Han, Dian‐sen Li, Lei Jiang, Dai‐ning Fang

**Affiliations:** ^1^ Key Laboratory of Bio‐Inspired Smppart Interfacial Science and Technology Ministry of Education School of Chemistry Beihang University Beijing 100191 China; ^2^ Beijing Advanced Innovation Center for Biomedical Engineering Beihang University Beijing 100191 China; ^3^ State Key Laboratory for Turbulence & Complex Systems College of Engineering Peking University Beijing 100871 China

**Keywords:** Co doping, flexible quasi‐solid‐state supercapacitor, potassium‐ion batteries, sodium‐ion batteries

## Abstract

Manganese dioxide (MnO_2_) is considered as a strong candidate in the field of new‐generation electronic equipment. Herein, Co‐MnO_2_ has excellent electrochemical properties in tests as the cathode electrode of sodium‐ion batteries and potassium‐ion batteries. The rate performance remains at 50.2 mAh g^−1^ at 200 mA g^−1^ for sodium‐ion batteries. X‐ray diffraction (XRD) is utilized to evaluate the crystal structure transition from Co_0.2_‐MnO_2_ to NaMnO_2_ with discharge to 1 V, proving that Co‐doping does indeed facilitate the acceleration of ion transport and support layer spacing to stabilize the structure of MnO_2_. Subsequently, highly conductive (0.0848 S cm^−1^) gel‐type supercapacitors are prepared by combining Co_0.2_‐MnO_2_, potassium hydroxide (KOH), and poly(vinyl alcohol) (PVA) together. Co_0.2_‐MnO_2_ provides capacitive behavior and strengthens the hydrogen bonds between molecules. KOH acts as an ion crosslinker to enhance hydrogen bond and as electrolyte to transport ions. 5 wt% Co_0.2_‐MnO_2_@KOH/PVA has superb mechanical endurance, appreciable electrical conductivity, and ideal capacitive behavior. The quasi‐solid‐state supercapacitor demonstrates stabilized longevity (86.5% at 0.2 mA cm^−3^ after 500 cycles), which can greatly promote the integration of flexible energy storage fabric devices.

## Graphical abstract

This paper reports the Na‐ion and K‐ion storage mechanism of Co_0.2_‐MnO_2_, and the mechanical property with capacitance of 5 wt% Co_0.2_‐MnO_2_@KOH/PVA. 5 wt% Co_0.2_‐MnO_2_@KOH/PVA has the largest tensile strength and compressive resistance. The high volume capacitances and energy density of quasi‐solid‐state supercapacitors provide direction for the next generation of wearable device applications.

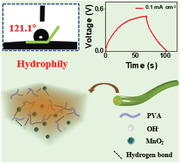

## 1 Introduction

Among various active materials used in sodium‐ion batteries (SIBs), potassium‐ion batteries (PIBs), and supercapacitors, manganese dioxide (MnO_2_) is a promising candidate due to its impressive theoretical specific capacity/capacitance, wide working window, and eco‐friendliness.^[^ ^1^ ^]^ Whereas, MnO_2_‐based materials exhibit serious performance degradation, which is affected by their low electronic conductivity (≈10^−5^ to 10^−6^ S cm^−1^), slow diffusion rate of ions, narrow tunnel size, and dramatic structural change during the charge/discharge process.^[^ ^2^ ^]^ Doping heteroatoms, on the one hand, can optimize the electronic structure of MnO_2_, thereby shortening ion transfer routes and improving its constitutive conductivity; on the other hand, can play a role in extending and supporting layer spacing to prevent structural collapse.^[^ ^3^ ^]^

Furthermore, MnO_2_‐based electrode materials with high electrical conductivity can be used to prepare flexible supercapacitors, which have tremendous potential in next generation wearable device applications due to its simple structural strategy, fast charge–discharge time, and long circular life.^[^ ^4^ ^]^ The key to construct a stretchable MnO_2_‐based supercapacitor is to combine an MnO_2_‐based electrode material with the elastomer material successfully. Hydrogels have been known for malleability and flexibility among various kinds of elastomer substrates.^[^ ^5^ ^]^ Poly(vinyl alcohol) (PVA), a kind of polymer, can be used to manufacture a nontoxic, biodegradable, and deformable matrix. However, there are many problems needed to be solved urgently, such as poor mechanics properties, and inferior ionic conductivity (10^−5^–10^−8^ S cm^−1^).^[^ ^6^ ^]^Actually, doping inorganic substances, like borax and FeCl_3_, can not only improve the conductivity, but also cross‐link the PVA to form a polymer network and then improve the mechanical properties.^[^ ^7^ ^]^

Herein, we report the Na‐ion and K‐ion storage mechanism of Co‐MnO_2_, and the mechanical property with capacitance of Co‐MnO_2_@KOH/PVA. Minutely, Co_0.2_‐MnO_2_ has a capacity of 71.8 mAh g^−1^ after 100 cycles in SIBs. A series of tests has been used to elucidate the electrochemical kinetics. The X‐ray diffraction (XRD) reveals that the characteristic peak of NaMnO_2_ appears. 5 wt% Co_0.2_‐MnO_2_@KOH/PVA has the largest tensile strength of 4.25 MPa and compressive resistance of 4.7 MPa as the strain increases to 70%. The volume capacitances of quasi‐solid‐state supercapacitors are 6.86, 6.44, 4.2, 3.1, and 1.8 mF cm^−3^ at current densities of 0.1, 0.2, 0.3, 0.5, and 1 mA cm^−3^.

## 2 Results and Discussion

### 2.1 Phase, Component, and Morphological Characterizations

**Figure 1:**
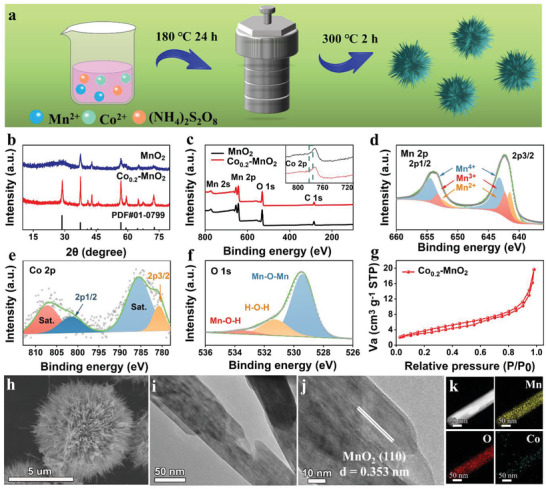
a) Schematic illustrating the fabrication process. b,c) XRD and XPS survey spectra of Co_0.2_‐MnO_2_ and MnO_2_. d–f) High‐resolution spectra of Mn 2p, Co 2p, and O 1s of Co_0.2_‐MnO_2_. g) Nitrogen adsorption–desorption isotherm, h) high‐magnification SEM images, i,j) TEM images, and k) EDS mapping images of Co_0.2_‐MnO_2_.

**Figure** **1**a demonstrates a schematic illustration that Co‐MnO_2_ was synthesized by a one‐step hydrothermal process. In order to explore the crystal structure and composition of Co_0.2_‐MnO_2_, X‐ray diffraction (XRD) and X‐ray photoelectron spectroscopy (XPS) spectra were performed. Figure 1b indicates the XRD patterns of Co_0.2_‐MnO_2_ and MnO_2_ nanorods. All diffraction peaks of Co_0.2_‐MnO_2_ agree well with MnO_2_ (PDF#01‐0799) without impurity peak. Compared with pure MnO_2_, Co_0.2_‐MnO_2_ shows a characteristic peak of (110) with a higher intensity and sharper peak shape, which confirms that Co not only supports and widens the layer spacing but also contributes to the exposure of a (110) crystal plane. Subsequently, XPS is used to explore the chemical compositions and valence states of Co_0.2_‐MnO_2_. The existence of Co can be proved by the appearance of Co 2p peaks in a full spectrum of Co_0.2_‐MnO_2_ (Figure 1c). As illustrated in the Figure 1d, the two spin orbitals of Mn 2p are grouped into three parts, standing for Mn^4+^, Mn^3+^, and Mn^2+^.^[^ ^8^ ^]^ The high‐resolution spectrum of Co 2p in Figure 1e exhibits two characteristic peaks of 801.6 and 780.7 eV, corresponding to the binding energies of Co 2p_1/2_ and Co 2p_3/2_.^[^ ^9^ ^]^ In the Figure 1f of O 1s spectrum, the peaks of 533.6, 531.4, and 529.5 eV are parallel to hydrous metallic oxides (Mn/Co─O─H), residual water molecule (H—O—H), and metallic oxides (Mn/Co—O—Mn/Co), respectively.^[^ ^10^ ^]^ These facts confirm that the element of Co is successfully doped into the crystal lattice of MnO_2_.

The morphology characterization can be authenticated by Brunauer–Emmett–Teller (BET), scanning electron microscopy (SEM), and transmission electron microscopy (TEM). The surface area of Co_0.2_‐MnO_2_ turns out to be 11.183 m^2^ g^−1^ in Figure 1g. In fact, the large specific surface area of the material can enhance the area for the electrolyte to penetrate. The low‐ and high‐magnification SEM images (Figure S1, Supporting Information, and Figure 1h) of Co_0.2_‐MnO_2_ nanorods show urchin‐like clusters with diameters of 2–10 um. Low‐magnification TEM image in Figure 1i further reveals that the diameter of nanorods is ≈70 nm, the enlarged part (Figure 1j) of which demonstrates evident lattice fringe of 0.353 nm, responding to the (110) plane of Co_0.2_‐MnO_2_. The energy‐dispersive spectroscopy (EDS) images (Figure 1k) show that Mn, O, and Co elements are evenly dispersed on each nanorod, displaying that Co is uniformly distributed in the MnO_2_ matrix.

### 2.2 Sodium/Potassium Storage Performance

**Figure 2:**
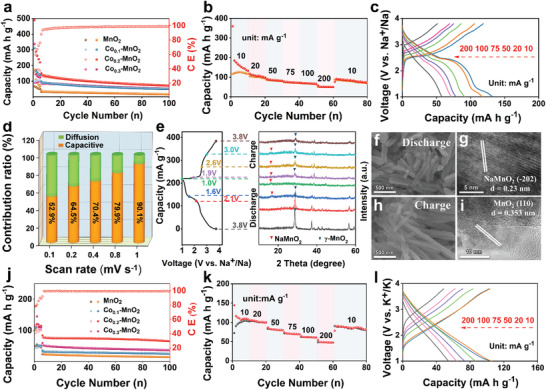
a) Cycling performance of different materials at a current density of 100 mA g^−1^, b) rate performance, and c) charge/discharge curves of Co_0.2_‐MnO_2_ at different current densities for SIBs. d) Percentage of pseudocapacitive contribution at different scan rates, e) XRD pattern of electrodes at different states, f,h) SEM, g,i) TEM images of the electrode at 1 and 3.8 V for SIBs, respectively. j) Cycling performance of different materials at a current density of 100 mA g^−1^, k) rate performance, and l) charge/discharge curves of Co_0.2_‐MnO_2_ at different current densities for PIBs.

The electrochemical performances of Co‐MnO_2_ cathode were analyzed by 2032 cells with the range of 1.0–3.8 V for SIBs. All electrodes in **Figure** **2**a are pre‐activated by 5 cycles at 10 mA g^−1^ and then cycle at a constant current density of 100 mA g^−1^. Co_0.2_‐MnO_2_ has a discharge capacity of 71.8 mAh g^−1^ after 100 cycles with a coulombic efficiency (CE) approaching 100%, whereas, Co_0.1_‐MnO_2_, Co_0.3_‐MnO_2_, and MnO_2_ show poor stability (50.5, 51.5, and 17.0 mAh g^−1^) throughout the charge–discharge cycles. The fact indicates that the doping of Co can significantly improve the electrochemical performance of MnO_2_‐based materials and Co_0.2_‐MnO_2_ possesses the best reversible capacity and structural stability than any other material.^[^ ^11^ ^]^ Figure 2b documents the rate data of 132.5, 98.5, 81.8, 71.7, 66.5, and 50.2 mAh g^−1^ with current densities of 10, 20, 50, 75, 100, and 200 mA g^−1^. The performance remains at 71.6 mAh g^−1^ when the current density backs to 10 mA g^−1^. Figure 2c exhibits charge/discharge curves of Co_0.2_‐MnO_2_ at different current densities. The similar curves prove the splendid reversibility and stability of Co_0.2_‐MnO_2_ once again. The performance of this work is better than that of other materials, the comparison diagram is shown in Figure S2, Supporting Information.^[^ ^12^ ^]^

To interpret the prominent electrochemical properties, the cyclic voltammetry (CV) curves (0.1 to 1.0 mV s^−1^) have been attained to elucidate the electrochemical kinetics. As shown in Figures S3 and S4, Supporting Information, the calculated slope values of specific peak currents are greater than 0.5, implying that the capacity is attributed to a combination of a diffusion and pseudocapacitive process.^[^ ^13^ ^]^ The pseudocapacitive contribution ratios in Figure 2d increase from 52.9% to 90.1% with a scan speed from 0.1 to 1.0 mV s^−1^, further verifying that the material has an excellent fast charging/discharging ability. In addition, Figure S5, Supporting Information, shows the Nyquist diagram of the impedance spectrum of electrodes with Co_0.1_‐MnO_2_, Co_0.2_‐MnO_2_, Co_0.3_‐MnO_2_, and undoped MnO_2_, as the active material. The values of charge‐transfer resistance (*R*_ct_) for Co_0.1_‐MnO_2_, Co_0.2_‐MnO_2_, Co_0.3_‐MnO_2_, and undoped MnO_2_ electrodes are 2645, 1571, 2586, and 4749 Ω, respectively. The smaller charge‐transfer resistance (*R*_ct_) in the high frequency region indicates that doping with Co enhances sodium intercalation near the electrode/electrolyte interface. The real part (*Z*_Re_) in the low‐frequency range is linear against the square root of the angular frequency (*ω* ^−1/2^, *ω* = 2*π*f) in Figure S6, Supporting Information. The slope (Warburg coefficient) of Co_0.2_‐MnO_2_ is the lowest, indicating its fast sodium ions diffusivity, which in turn illustrates the best rate performance. Just as we predicted, the doping of Co reorganizes the electronic distribution of MnO_2_, promoting the Na^+^ adsorption and the reaction kinetics.

In addition to investigating the kinetic properties of SIBs, the redox peak of the CV diagram (Figure S7, Supporting Information) and the ex‐situ XRD (Figure 2e) can be used to explore the crystal structure transition of Co_0.2_‐MnO_2_ electrodes at different states after 10 cycles. It can be seen that the characteristic peaks of NaMnO_2_ (PDF#25‐0845) appear and the characteristic peaks of MnO_2_ gradually decrease with discharge to 1 V. The SEM in Figure 2f and TEM in Figure 2g further confirm the structural stability and presence of NaMnO_2_. The peaks of MnO_2_ will reappear when charging to 3.8 V, as shown in Figure 2h,i.

In fact, Co_0.2_‐MnO_2_ not only has an excellent performance in SIBs, but also has outstanding results in PIBs. As shown in Figure 2j, the capacity still maintains at 65.9 mAh g^−1^ after 100 cycles at 100 mA g^−1^. The rate performance in Figure 2k manifests that Co_0.2_‐MnO_2_ cathode can stay at 47.6 mAh g^−1^ at 200 mA g^−1^. The CV curves in Figure 2i are analogous at different current densities, which also proves the stability and reversibility of the material. As previously speculated, Co‐doping does help to speed up ion transport and support layer spacing to stabilize the structure of MnO_2_.

### 2.3 Performance of Co‐MnO_2_@KOH/PVA Fiber Hydrogels

As is well‐known, supercapacitor is a new type of energy storage device with the characteristics of the energy storage mechanism of electrochemical cells and fast charge/discharge. Co_0.2_‐MnO_2_, with stable structure and fast transmission speed, could also be used to make supercapacitors. Flexible supercapacitors can be prepared by combining elastic material with manganese dioxide. Poly(vinyl alcohol) (PVA, thickener) is a water‐soluble polymer, containing a mass of hydroxyl groups. KOH, as crosslinking agents, can enhance the mechanical properties of PVA by increasing the extent of intermolecular cross‐linking and the extent of network entanglement.^[^ ^14^ ^]^ As shown in Figures S9–S11, Supporting Information, the stress, fracture energy, and elongation at break increase with the increase of KOH content, which phenomenon is caused by the augment of hydrogen bond. Hydrogen bond (OH groups) can be verified by the Fourier transfer infrared spectroscopy (FTIR) spectra in Figure S12, Supporting Information. The peaks of *V*_O‐H_ shift from 3282 to 3255 cm^−1^ of 1 m, 2 m, and 2.5 m KOH‐PVA, indicating the hydrogen bonds are gradually increasing.^[^ ^15^ ^]^ The pictures of these four materials (Figure S13, Supporting Information) can also prove the above point. The hydrogel gradually becomes transparent and strong as KOH increases. However, the elastic modulus does not conform to this law. The hydrogen bond is too strong to be stretched and has inferior rebound resilience, resulting in that the elastic modulus of 2.5 m KOH‐PVA is weaker than that of 2 m KOH‐PVA. Therefore, 2 m KOH‐PVA is used for the next test.

**Figure 3:**
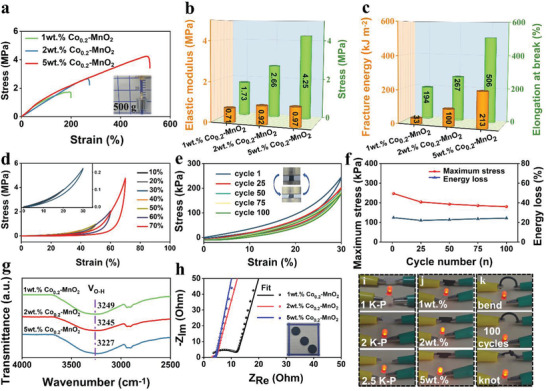
a) Tensile stress–strain curves, b) elastic modulus and stress, c) fracture energy and elongation at break of 1 wt% Co_0.2_‐MnO_2_@KOH/PVA, 2 wt% Co_0.2_‐MnO_2_@KOH/PVA, and 5 wt% Co_0.2_‐MnO_2_@KOH/PVA. d) Compression properties, e) cyclic compression performance at 30% strain, f) maximum stress and energy loss of 5 wt% Co_0.2_‐MnO_2_@KOH/PVA. g) FT‐IR spectra for 2500–4000 cm^−1^, h) Nyquist plots of 1 wt% Co_0.2_‐MnO_2_@KOH/PVA, 2 wt% Co_0.2_‐MnO_2_@KOH/PVA, and 5 wt% Co_0.2_‐MnO_2_@KOH/PVA. i–k) The circuit experiment of PVA, 1 m KOH, 2 m, 2.5 m KOH, 1 wt% Co_0.2_‐MnO_2_@KOH/PVA, 2 wt% Co_0.2_‐MnO_2_@KOH/PVA, and 5 wt% Co_0.2_‐MnO_2_@KOH/PVA.

Similarly, the mechanical properties of 2 m KOH‐PVA can be adjusted readily by doping different masses of Co_0.2_‐MnO_2_. **Figure** **3**a–c compare the tensile properties of 1 wt% Co_0.2_‐MnO_2_, 2 wt% Co_0.2_‐MnO_2_, and 5 wt% Co_0.2_‐MnO_2_@KOH/PVA. 10 wt% Co_0.2_‐MnO_2_@KOH/PVA is too crosslinked to form a flexible material to participate in the test. The largest tensile strength reaches 4.25 MPa of 5 wt% Co_0.2_‐MnO_2_@KOH/PVA, which is 26 times more than that of pure PVA. 5 wt% Co_0.2_‐MnO_2_@KOH/PVA with the diameter of 3 mm can pull up to 500 g of weight in the illustration of Figure 3a. Not only that, elastic modulus, fracture energy, and elongation at break are also the largest with the value of 0.97 MPa, 213 kJ m^−2^, and 506%, demonstrating 5 wt% Co_0.2_‐MnO_2_@KOH/PVA has the most excellent tensile properties.

In fact, the compressibility of 5 wt% Co_0.2_‐MnO_2_@KOH/PVA is impressive. It can be seen in Figure 3d that the stress increases to 4.7 MPa as the strain increases to 70%, and the curves overlap well at the strain of 10–30%, indicating the material has outstanding compressive resistance. In order to verify cyclic compression stability, 100 compression cycles were performed on 5 wt% Co_0.2_‐MnO_2_@KOH/PVA in Figure 3e. The curves of 25th, 50th, 75th, and 100th cycles are roughly coincident. Subsequently, Figure 3f reports that the calculated data of maximum stress and energy loss are 247 kPa and 24% at the first compression, and then tend to be stable after several cycles. The key to excellent mechanical properties of materials is the degree of cross‐linking between molecules, known as hydrogen bonds. The peaks of the hydrogen bond in Figure 3g shift from 3249 to 3227 cm^−1^, indicating that the hydrogen bond is strengthened with the increase of Co_0.2_‐MnO_2_. In conclusion, it shows that 5 wt% Co_0.2_‐MnO_2_@KOH/PVA has excellent mechanical durability, which provides a guarantee for application in flexible supercapacitors.

It is also crucial that the material has admirable electrical conductivity. In order to verify that, the electrochemical workstation was used to test the resistance of the material in Figure 3h and Figures S15 and S16, Supporting Information, and we calculated the electroconductibility value (Table S1, Supporting Information). The results in Table S1, Supporting Information, show that both the addition of KOH and Co_0.2_‐MnO_2_ can promote the conductivity of PVA, and it will increase with the increase of KOH and Co_0.2_‐MnO_2_. The conductivity is the highest (0.0848 S cm^−1^), when the content of Co_0.2_‐MnO_2_ is 5 wt% and KOH is 2 m. Moreover, it is obvious from the circuit experiment (Figure 3i–k) that the brightest light‐emitting diode (LED), as a conductive connector, is lit by 5 wt% Co_0.2_‐MnO_2_@KOH/PVA, indicating that its conductivity is the highest. And even more interesting, the LED can be bright even when the 5 wt% Co_0.2_‐MnO_2_@KOH/PVA gel fiber is bent, stretched 100 times, and knotted. This shows that the conductive network of the material is stable and is not disturbed by external forces. Moreover, the comparison of performance between the Co_0.1_‐MnO_2_, Co_0.2_‐MnO_2_, and Co_0.3_‐MnO_2_ in hydrogel supercapacitors has been carried out in Figure S17, Supporting Information, and the results show that Co_0.2_‐MnO_2_ still performs well in hydrogel supercapacitors.

**Figure 4:**
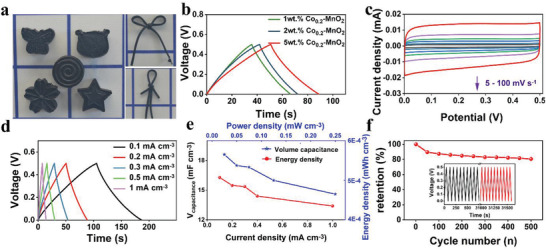
a) Flexible display of materials. b) GCD curves of 1 wt% Co_0.2_‐MnO_2_@KOH/PVA, 2 wt% Co_0.2_‐MnO_2_@KOH/PVA, and 5 wt% Co_0.2_‐MnO_2_@KOH/PVA. c) CV curves at scan rates ranging from 5 to 100 mV s^−1^ of d) GCD curves at different current densities of e) volume capacitances calculated based on the GCD curves and volume energy density versus power density of f) cycle life at 0.2 mA cm^−3^ of 5 wt% Co_0.2_‐MnO_2_@KOH/PVA, the inset is the first ten and the last ten GCD curves.

As shown in **Figure** **4**a, the 5 wt% Co_0.2_‐MnO_2_@KOH/PVA can be prepared in a variety of shapes, and the prepared fibers can be bent and folded, which demonstrates the plasticity of the material and can be further used to make flexible supercapacitors. In order to select the material with the best electrochemical properties, a three‐electrode system was applied in 1 m KOH solution to evaluate the electrochemical behavior of three different yarn electrodes (Figure 4b). 5 wt% Co_0.2_‐MnO_2_@KOH/PVA has the largest galvanostatic charge/discharge (GCD) curves area, that is, has the highest specific capacitance. The reason is that on the one hand, Co_0.2_‐MnO_2_ owns the excellent capacitive performance, and on the other hand, it has the highest content of Co_0.2_‐MnO_2_ in 5 wt% Co_0.2_‐MnO_2_@KOH/PVA fiber. Figure 4c shows the rectangular CV curves ranging from 5 to 100 mV s^−1^ of 5 wt% Co_0.2_‐MnO_2_@KOH/PVA, revealing ideal capacitance characteristics. In addition, a 5 wt% Co_0.2_‐MnO_2_@KOH/PVA electrode still maintains a rectangle for all CV curves at low rates, indicating that the fiber has fast electronic transfer.^[^ ^16^ ^]^ The GCD curves of 5 wt% Co_0.2_‐MnO_2_@KOH/PVA at different current densities in Figure 4d is triangular‐like and has excellent symmetry, revealing ideal capacitance behavior. According to the calculation in Figure 4e, the volume capacitances are 16.3, 15.5, 15.4, 14.4, and 13.4 mF cm^−3^ at 0.1, 0.2, 0.3, 0.5, and 1 mA cm^−3^. The energy densities of material are 0.56, 0.54, 0.53, 0.50, and 0.46 μWh cm^−3^ when the power densities are 0.025, 0.05, 0.075, 0.125, and 0.25 mW cm^−3^. As shown in Figure 4f, the capacitance retention is 80.5% over 500 cycles, revealing excellent electrochemical stability.

**Figure 5:**
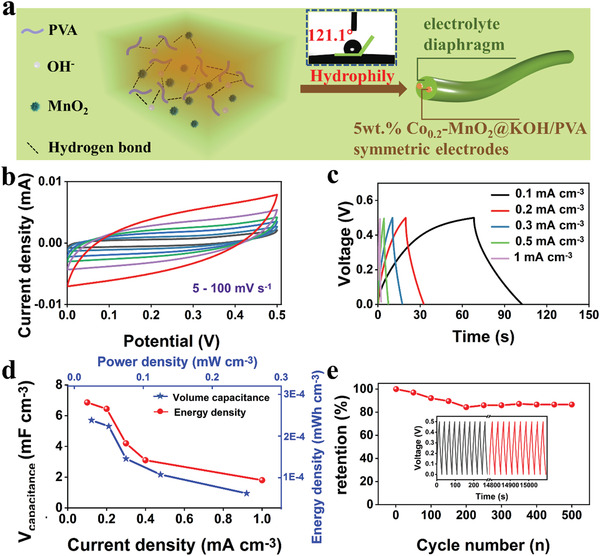
a) Schematic diagram, b) CV curves ranging from 5 to 100 mV s^−1^ of c) GCD curves of d) volume capacitances calculated based on the GCD curves and volume energy density versus power density of e) cycle life at 0.2 mA cm^−3^ of quasi‐solid‐state supercapacitor, the inset is the first ten and the last ten GCD curves.

Subsequently, a pair of 5 wt% Co_0.2_‐MnO_2_@KOH/PVA gel fibers were encapsulated parallelly in 1 m KOH‐PVA acting as electrolyte and separator to evaluate the electrochemical properties of a quasi‐solid‐state asymmetric supercapacitor in **Figure** **5**a. The hydrogen bond between PVA, OH^−^, and Co_0.2_‐MnO_2_ gives the material robust flexibility to facilitate its stretching and bending. The contact angle is greater than 120° (Figure 5a), indicating the hydrophilicity of the material. The CV curves in Figure 5b of the supercapacitor are rectangular‐shaped with the scan rates from 5 to 100 mV s^−1^, indicating favorable reversibility and rate capability. GCD curves in Figure 5c are a symmetric triangle, indicating unobstructed charge transport between the two yarns. In Figure 5d, the volume capacitances are 6.86, 6.44, 4.2, 3.1, and 1.8 mF cm^−3^ at current densities of 0.1, 0.2, 0.3, 0.5, and 1 mA cm^−3^. The energy densities of material are 0.24, 0.23, 0.15, 0.11, and 0.06 μWh cm^−3^ when the power densities are 0.025, 0.05, 0.075, 0.125, and 0.25 mW cm^−3^. As shown in Figure 5e, the supercapacitor demonstrates stabilized longevity: 86.5% of its initial capacitance is retained after 500 cycles of GCD measurements at 0.2 mA cm^−3^, which provides a solid candidate for building transparent and flexible supercapacitors.

## 3 Conclusion

In summary, Co substitution can improve the electron transport, increase the intrinsic conductivity of MnO_2_, and stabilize the interlayer to inhibit the collapse of structure, effectively. Co_0.2_‐MnO_2_ exhibits a splendid capacity at a current density of 100 mA g^−1^ (71.8 mAh g^−1^ after 100 cycles for SIBs, 65.9 mAh g^−1^ after 100 cycles for PIBs). Afterwards, 5 wt% Co_0.2_‐MnO_2_ combining with 2 m KOH‐PVA has excellent mechanical stability and conductivity to be used in flexible electronic devices. Quasi‐solid‐state supercapacitors possess superior energy densities and a stable life of 86.5%, which provides direction for the next generation of wearable device applications.

## 4 Experimental Section

### Materials

Manganese sulfate, monohydrate (MnSO_4_·H_2_O, Macklin, 99.9%), ammonium persulfate ((NH_4_)_2_S_2_O_8_, Macklin, 99.0%), cobalt nitrate, hexahydrate (Co(NO_3_)_2_·6H_2_O, Macklin, 99%), poly(vinyl alcohol) 1799 (PVA, Macklin, alcoholysis degree: ≈98–99%), and potassium hydroxide (KOH, Macklin, 90%), all reagents were used directly without purification.

### Preparation of Co‐MnO_2_

0.01 mol MnSO_4_·H_2_O, 0.01 mol (NH_4_)_2_S_2_O_8_, and the proper amount of Co(NO_3_)_2_·6H_2_O (the molar ratios of Mn:Co are 10:1, 5:1, and 10:3) were dissolved in deionized water and then stirred 10 min at 20 ℃ to form a black solution. Next, the solution was transferred to a Teflon‐lined autoclave at 180 ℃ for 24 h. Finally, the Co‐MnO_2_ was gathered by filtration and washing it several times, followed by a calcining process at 300 ℃ for 2 h. According to the different content of Co(NO_3_)_2_·6H_2_O, the materials were named Co_0.1_‐MnO_2_, Co_0.2_‐MnO_2_, Co_0.3_‐MnO_2_ in turn.

### Preparation of Co‐MnO_2_@KOH/PVA Fiber Hydrogels

Co_0.2_‐MnO_2_ with different mass fractions (1, 2, and 5 wt%) were dispersed in 20 mL deionized water, in which PVA powders were added at 90 ℃ and stirred for 2 h (12%, w/v%). Next, different concentrations of KOH (1, 2, and 2.5 m) were added with intense agitation to form a viscous liquid. Finally, the liquid was injected into a silicone tube with a diameter of 3 mm, followed by three cycles of freeze‐thawing (−18 ℃ for 5 h and at 25 ℃ for 2 h), called 1 wt% Co_0.2_‐MnO_2_@KOH/PVA, 2 wt% Co_0.2_‐MnO_2_@KOH/PVA, and 5 wt% Co_0.2_‐MnO_2_@KOH/PVA. In addition, PVA hydrogels (12 wt%) with different concentrations of KOH (1, 2, and 2.5 M) were prepared, named 1 M KOH/PVA, 2 M KOH/PVA, and 2.5 M KOH/PVA, successively.

### Preparation of Fiber Supercapacitor

Two fibers (2 m KOH, 5 wt% Co_0.2_‐MnO_2_, diameter of 1 mm) were paralleled together and then coated with PVA electrolyte (PVA 12 wt%, 1 m KOH) to form fiber hydrogel supercapacitors (diameter of 3 mm).

### Electrochemical Tests

Electrochemical performance of SIBs/PIBs was tested by CR2032 cells with Na/K metal foil as the counter electrode and glass fiber (GF/F) as separator. The working electrode was obtained from a mixture of active materials, super P and PVDF (weight ratio: 7:2:1), coated onto Al foil, and then dried at 60 ℃ overnight. The corresponding electrolyte was 1 m Na_2_SO_4_ in ethylene carbonate (EC)/dimethyl carbonate (DEC) (1:1, volume%) with an addition of 2% fluoroethylene carbonate (FEC), and 1 m KFSI in ethylene carbonate (EC)/dimethyl carbonate (DEC) (1:1, volume%) for SIBs and PIBs, respectively. Cycling and rate performance were tested by LAND CT‐2001A battery‐test instrument in a range of 1–3.8 V. CHI660E (China Brilliance Shanghai) electrochemical workstation tested cyclic voltammetry (CV) and electrochemical impedance spectroscopy measurement (0.01 Hz to 100 kHz). The calculation formula of pseudocapacitance: the correlation between peak current (*I*) and scan rate (*v*): *i*  =  *av^b^*. The capacitance‐ and the diffusion‐control contribution can be distinguished by: *i* (V) = *k*_1_ *v* + *k*_2_ *v* ^1/2^.

The diffusion coefficient of Na^+^ (D_Na+_) could be decided by: $D_{\mathrm{Na}+}=0.5R^{2}T^{2}/A^{2}n^{4}F^{4}C^{2}\sigma^{2}\cdot R,T,A,n,F$, and C are constants, it can be seen that *σ* is inversely proportional to the diffusion coefficient (D_Na+_). *σ* (Warburg coefficient) can be determined by the slope of the fitting linear of *Z*_Re_‐*ω* ^−1/2^:*ω*  =  2Π*f*, *Z*_Re_ =  *R* + *σω* ^−1/2^.^[^ ^17^ ^]^

For supercapacitor, CV, electrochemical impedance spectroscopy and galvanostatic charge–discharge measurements of hydrogels were performed by using an CHI660E electrochemical workstation (−0.2–0.4 V). The volume capacitance (*C*_v_) was computed by the equation: *C*_V_ =  *I*  ×  t  ×  *U* ^−1^  ×  *V* ^−1^. *I*, *t*, *U*, *V* stand for the current, discharge time, potential window, and the volume of the Co‐MnO_2_@KOH/PVA fiber hydrogels, severally.^[^ ^18^ ^]^ The volume energy density (*E*) and volume power density (*P*) were estimated by the following equation: *E*  =  C*U* ^2^  ×  2^−1^  ×  3600^−1^ and *P*  =  3600E  ×  *t* ^−1^.^[^ ^19^ ^]^

### Characterizations

XRD (Shimadzu XRD‐6000) was used to characterize the crystal structure of the Co‐MnO_2_ powder. SEM (JEOL JSM‐7500F) and TEM (FEI TalosF200x) were carried out to characterize the morphology and structural characteristics of Co‐MnO_2_ powder. XPS was applied to analyze the valence of Mn, O, C, and Co elements. Specific surface area analyzer (Mike 2020 HD88) was used to survey Brunner–Emmet–Teller (BET) surface area. spectrophotometer (BRUKER, ALPHA II) was applied to explore the Fourier transfer infrared spectroscopy (FTIR) spectra of the hydrogels. Mechanical testing machine (AGS‐X 1kN) was applied to the compression test and tensile test of the hydrogels.

## Conflict of Interest

The authors declare no conflict of interest.

## Supporting information

Supporting Information

## Data Availability

Research data are not shared.
