# Supplementary material for: Co‐MnO2 Nanorods for High‐Performance Sodium/Potassium‐Ion Batteries and Highly Conductive Gel‐Type Supercapacitors
Source: Adv Sci (Weinh). 2022 Jan 27;9(9):2105510. doi: 10.1002/advs.202105510 (PMC8948560; doi:10.1002/advs.202105510)
Supplement: Supplementary file 1 — Supporting Information [file ADVS-9-2105510-s001.pdf]

ADVANCED SCIENCE
Open Access

Supporting Information

for *Adv. Sci.*, DOI 10.1002/advs.202105510

Co-MnO_2 Nanorods for High-Performance Sodium/Potassium-Ion Batteries and Highly Conductive Gel-Type Supercapacitors

Jun Han, Dian-sen Li, Lei Jiang and Dai-ning Fang*

ADVANCED SCIENCE Open Access

Supporting Information

for *Adv. Sci.,* DOI: 10.1002/advs.202105510

Co-MnO_2 Nanorods For High-performance Sodium/Potassium-ion Batteries and Highly Conductive Gel-type Supercapacitors

Jun Han[a], *Dian-sen Li*[a,b,*], *Lei Jiang*[a], *Dai-ning Fang*[c]

[a] Key Laboratory of Bio-Inspired Smppart Interfacial Science and Technology, Ministry of Education, School of Chemistry, Beihang University, Beijing 100191, China

[b] Beijing Advanced Innovation Center for Biomedical Engineering, Beihang University, Beijing 100191, China

[c] State Key Laboratory for Turbulence & Complex Systems, College of Engineering, Peking University, Beijing, 100871, China

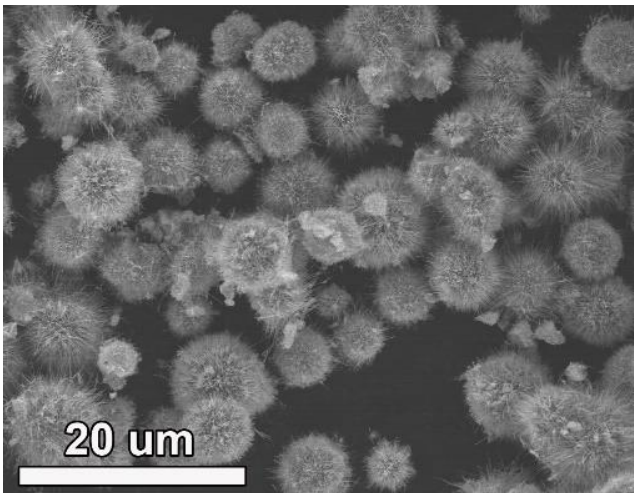

Figure S1. low-magnification SEM micrograms of $Co_{0.2}$-MnO_2.

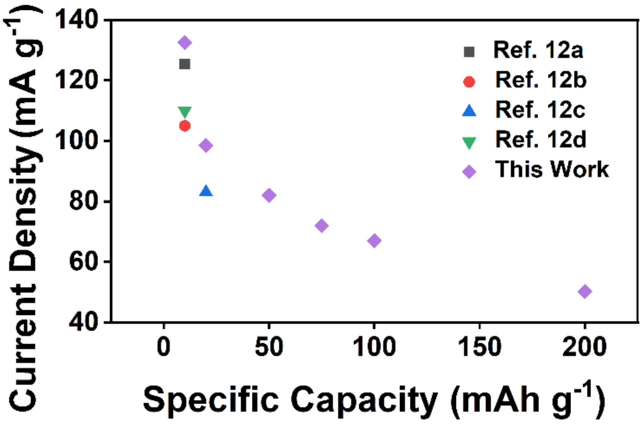

Figure S2. Comparison of performance of reported cathode materials for SIBs.

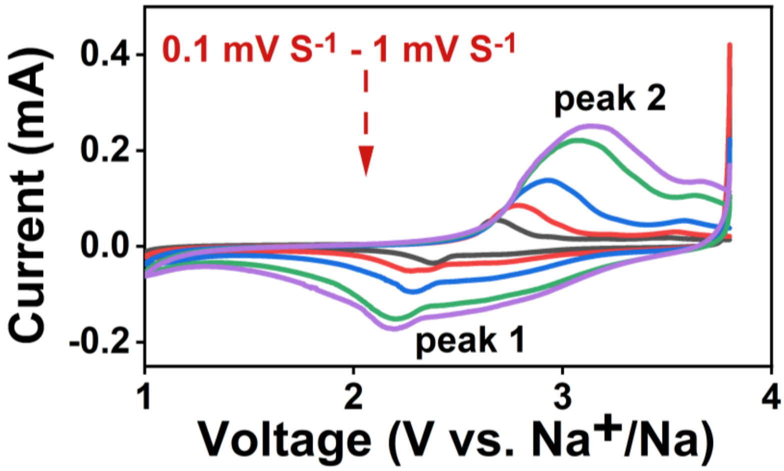

Figure S3. CV curves of $Co_{0.2}$-MnO_2 at different scan rates.

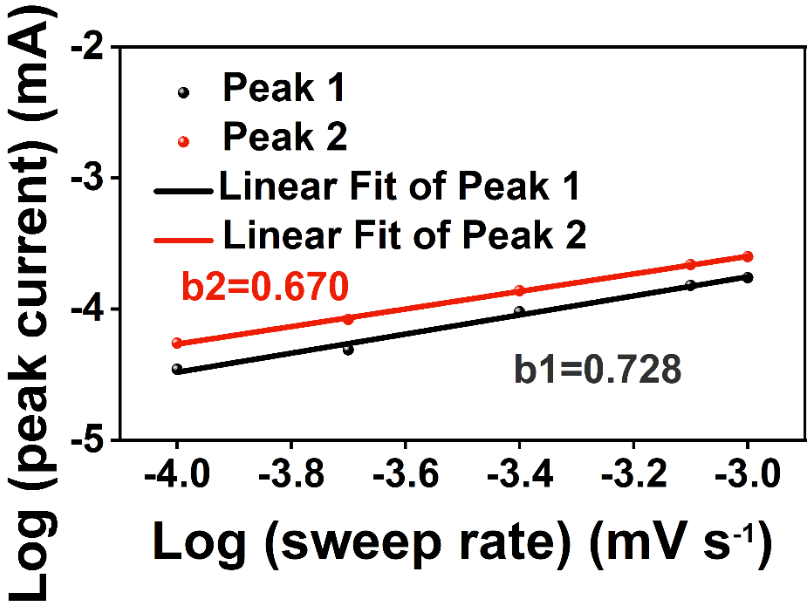

Figure S4. Log (current, mA) versus log (sweep rate, mV s^{-1}) plots at specific peak currents.

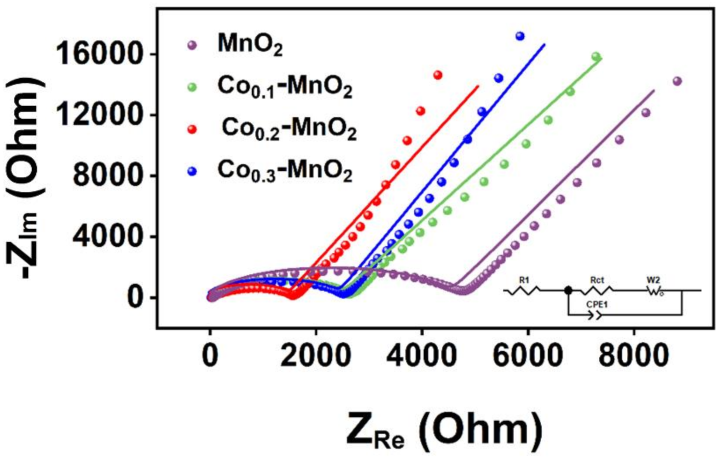

Figure S5. Nyquist curves of the electrode, (f) the fitted lines of the Z real vs. ω−1/2 in the low-frequency range.

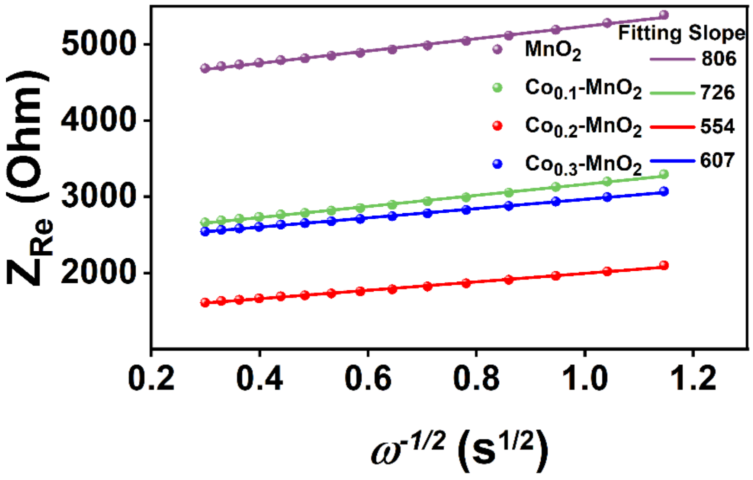

Figure S6. the fitted lines of the Z_{Re} real vs. $\omega^{-1/2}$ in the low-frequency range.

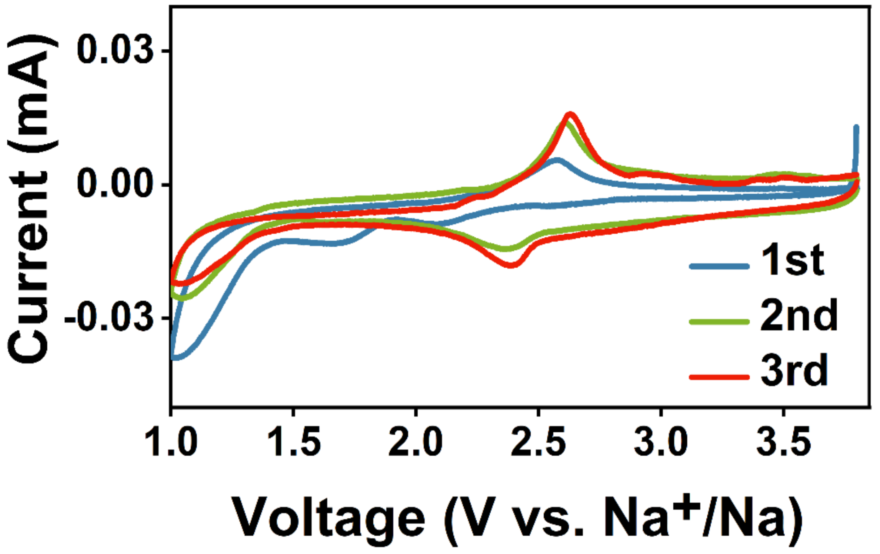

Figure S7. CV curve at 0.1 mV s^{-1} with a potential range of 1-3.8 V.

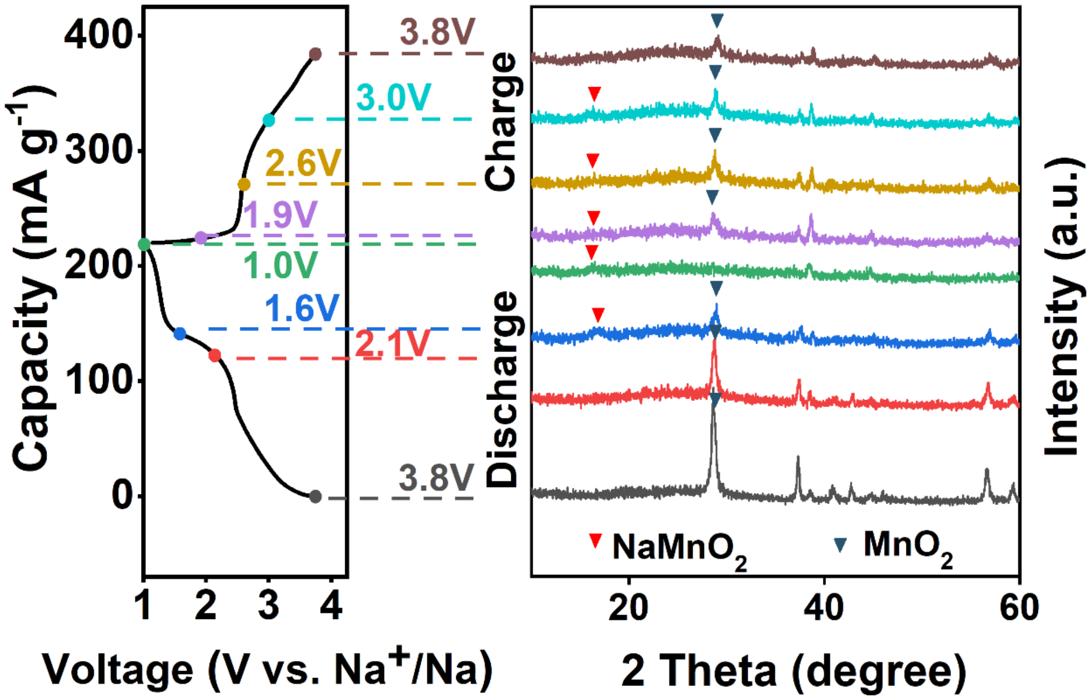

Figure S8. Ex-situ XRD pattern of Co-MnO_2 electrodes at different states.

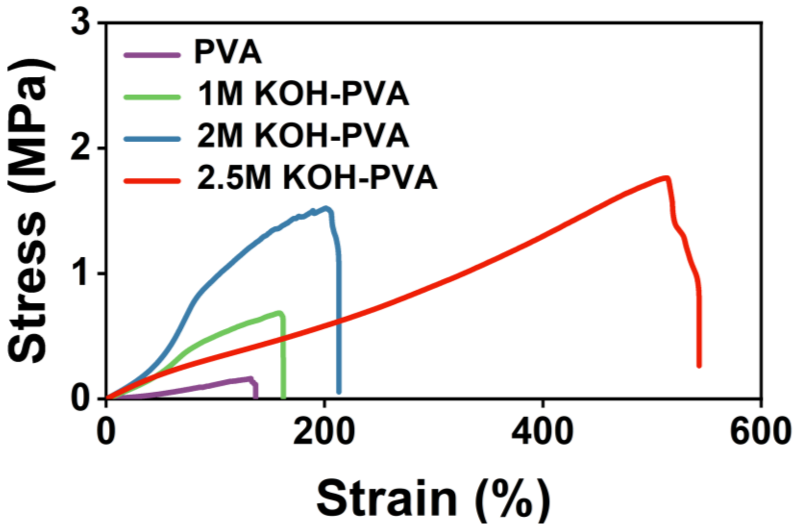

Figure S9. Tensile stress-strain curves of PVA, 1 M KOH-PVA, 2 M-PVA and 2.5 M KOH-PVA.

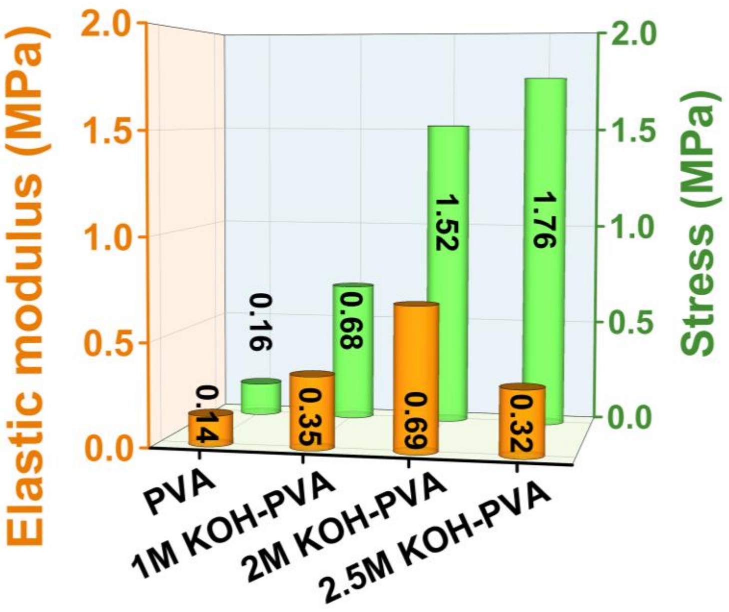

Figure S10. Elastic Modulus and stress of PVA, 1 M KOH-PVA, 2 M-PVA and 2.5 M KOH-PVA.

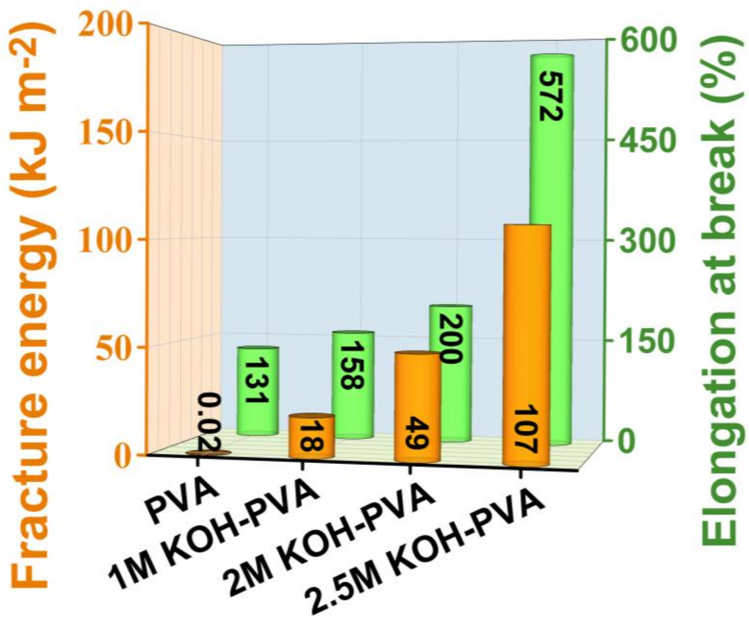

Figure S11. Fracture energy and Elongation at break of PVA, 1 M KOH-PVA, 2 M-PVA and 2.5 M KOH-PVA.

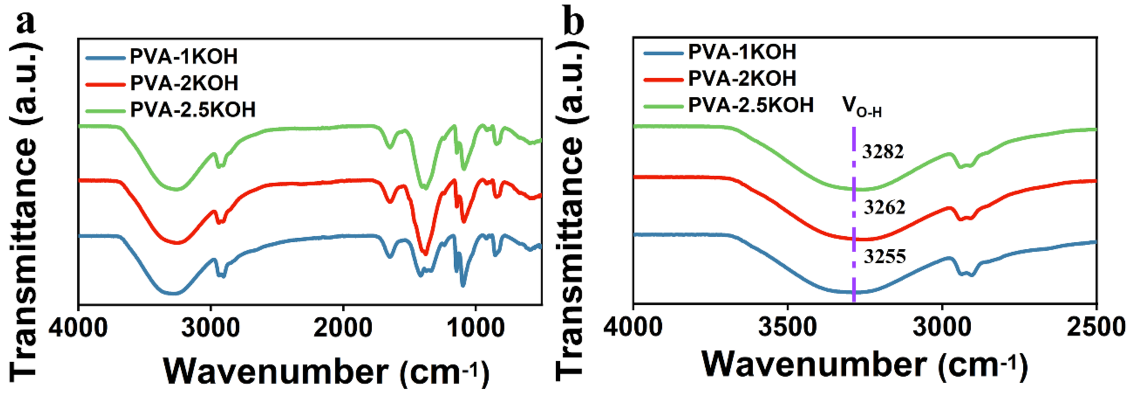

Figure S12. a) FT-IR spectra b) 2500-4000 cm^{-1} of 1 M KOH, 2 M and 2.5 M KOH-PVA.

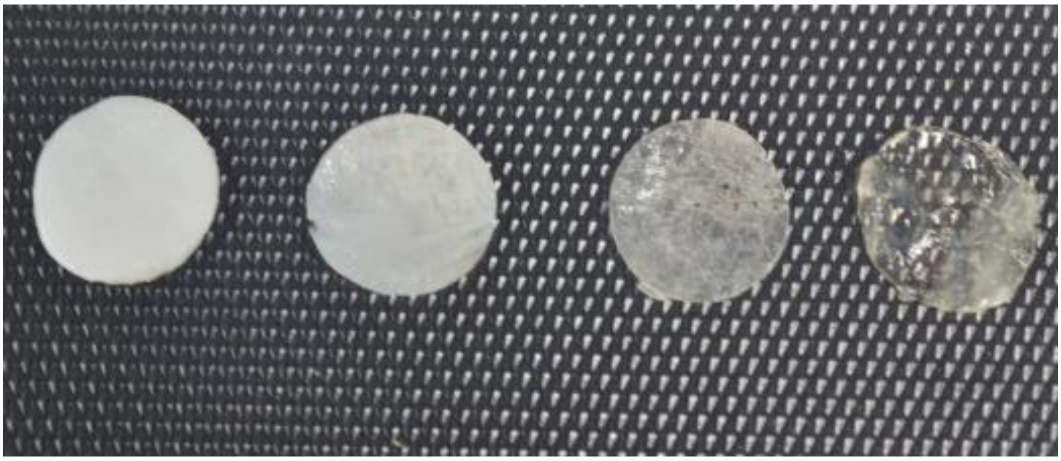

Figure S13. The picture of PVA, 1 M KOH, 2 M and 2.5 M KOH-PVA.

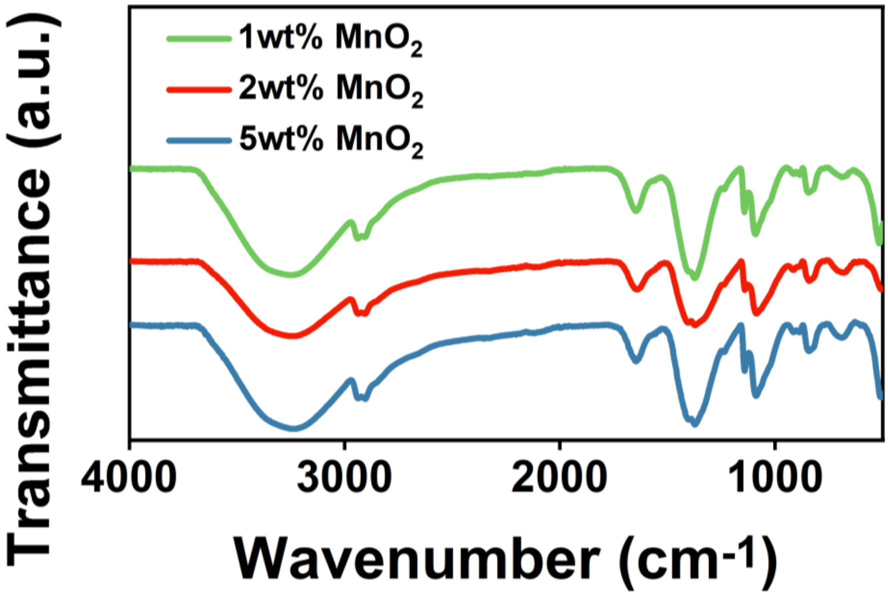

Figure S14. FT-IR spectra of 1Co-MnO_2@KOH/PVA, 2Co-MnO_2@KOH/PVA, 5Co-MnO_2@KOH/PVA.

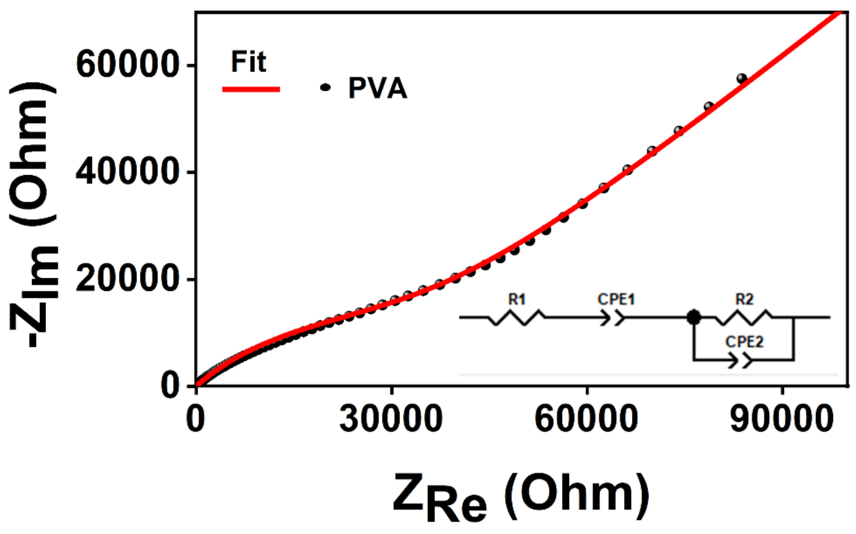

Figure S15. Nyquist plots for PVA.

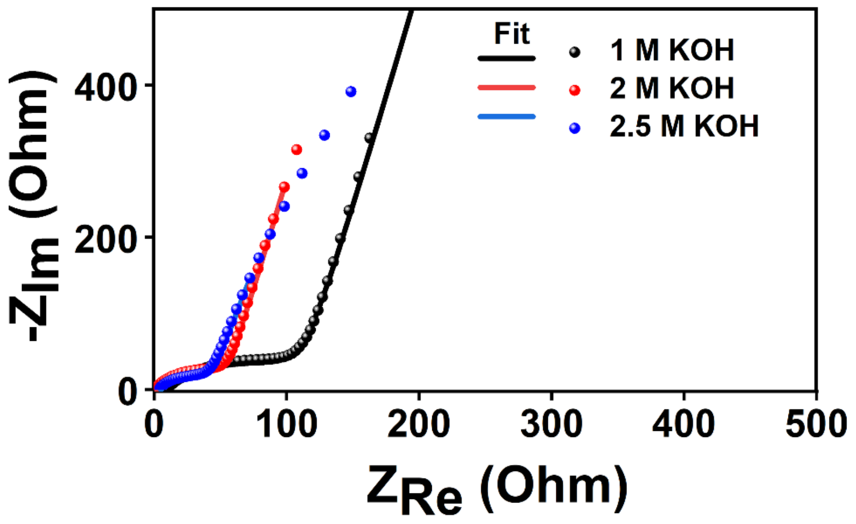

Figure S16. Nyquist plots for 1 M KOH, 2M and 2.5 M KOH.

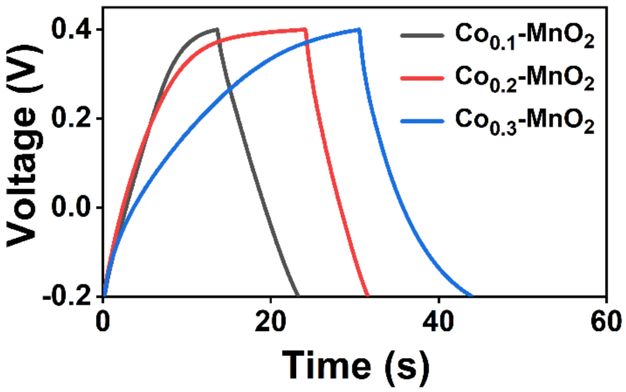

Figure S17. Comparison of performance between the $Co_{0.1}$-MnO_2, $Co_{0.2}$-MnO_2 and $Co_{0.3}$-MnO_2 in hydrogel supercapacitors.

Table S1. Electrical conductivity for PVA, 1 M KOH, 2M and 2.5 M KOH, 1Co-MnO_2@KOH/PVA, 2Co-MnO_2@KOH/PVA, 5Co-MnO_2@KOH/PVA

	**PVA**	**1M KOH**	**2M KOH**	**2.5M KOH**	**1wt% MnO_2**	**2wt% MnO_2**	**5wt% MnO_2**
**Area （cm^2）**	2	2	2	2	2	2	2
**thickness （cm）**	0.092	0.084	0.066	0.071	0.131	0.076	0.076
**resistance （Ω）**	30452	101.2	52.11	36.81	10	1.128	0.448
**electrical conductivity （S/cm）**	0.00000151	0.000415	0.00063	0.000964	0.00655	0.0337	0.0848

References:

[1] a) Q. Y. Shen, X. D. Zhao, Y. C. Liu, Y. P. Li, J. Zhang, N. Zhang, C. G. Yang, J. Chen, *Adv. Sci.* **2020**, 7, 2002199; b) S. M. Kang, D. Kim, K. S. Lee, M. S. Kim, A. H. Jin, J. H. Park, C. Y. Ahn, T. Y. Jeon, Y. H. Jung, S. H. Yu, J. Mun, Y. E. Sung, *Adv. Sci.* **2020**, 7, 2001263; c) Y. M. Li, Z. Z. Yang, S. Y. Xu, L. Q. Mu, L. Gu, Y. S. Hu, H. Li, L. Q. Chen, *Adv. Sci.* **2015**, 2, 1500031; d) S. Y. Xu, J. P. Wu, E. Y. Hu, Q. H. Li, J. N. Zhang, Y. Wang, E. Stavitski, L. W. Jiang, X. H. Rong, X. Q. Yu, W. L. Yang, X. Q. Yang, L. Q. Chen, Y. S. Hu, *J. Mater. Chem. A* **2018**, 6, 20795.
